# Reduced Crowding and Poor Contour Detection in Schizophrenia Are Consistent with Weak Surround Inhibition

**DOI:** 10.1371/journal.pone.0060951

**Published:** 2013-04-09

**Authors:** Valentina Robol, Marc S. Tibber, Elaine J. Anderson, Tracy Bobin, Patricia Carlin, Sukhwinder S. Shergill, Steven C. Dakin

**Affiliations:** 1 Department of General Psychology, University of Padua, Padua, Italy; 2 Institute of Ophthalmology, University College London, London, United Kingdom; 3 Institute of Cognitive Neuroscience, University College London, London, United Kingdom; 4 King’s College London, Institute of Psychiatry, London, United Kingdom; CNRS - Université Claude Bernard Lyon 1, France

## Abstract

**Background:**

Detection of visual contours (strings of small oriented elements) is markedly poor in schizophrenia. This has previously been attributed to an inability to group local information across space into a global percept. Here, we show that this failure actually originates from a combination of poor encoding of local orientation and abnormal processing of visual context.

**Methods:**

We measured the ability of observers with schizophrenia to localise contours embedded in backgrounds of differently oriented elements (either randomly oriented, near-parallel or near-perpendicular to the contour). In addition, we measured patients’ ability to process local orientation information (i.e., report the orientation of an individual element) for both isolated and crowded elements (i.e., presented with nearby distractors).

**Results:**

While patients are poor at detecting contours amongst randomly oriented elements, they are proportionally less disrupted (compared to unaffected controls) when contour and surrounding elements have similar orientations (near-parallel condition). In addition, patients are poor at reporting the orientation of an individual element but, again, are less prone to interference from nearby distractors, a phenomenon known as visual crowding.

**Conclusions:**

We suggest that patients’ poor performance at contour perception arises not as a consequence of an “integration deficit” but from a combination of reduced sensitivity to local orientation and abnormalities in contextual processing. We propose that this is a consequence of abnormal gain control, a phenomenon that has been implicated in orientation-selectivity as well as surround suppression.

## Introduction

Convergent evidence from psychophysics, electrophysiology and functional brain imaging indicates that patients with schizophrenia (SZ) exhibit persistent deficits in visual processing (for review see [1]). These patients show poorer detection of low compared to high spatial frequency (SF) gratings (for review see [2], [3]), a finding that is mirrored in patients exhibiting noisier visual evoked potentials (VEPs) in response to low SF stimuli [4], [5]. Such findings are often attributed to a selective deficit in the magnocellular visual pathway (although see e.g. [6]).

Another way in which contrast-processing differs in SZ is in the effect of *context*. Dakin et al. [7] showed that the dramatic reduction in perceived contrast of a target-patch that occurs when it is embedded in a high contrast background [8] is greatly reduced in patients with SZ. That patients are less prone to this centre-surround illusion (i.e. they perform *better* than matched controls) allows one to be confident that this is a consequence of a particular mechanism, rather than poorer performance, which could reflect a more generalised, e.g. attentional, deficit. Dakin et al. [7] interpreted this finding as a manifestation of decreased *gain control*, the inhibitory cortical processes that allow neurons to optimise their limited operating range. The inhibitory mechanisms linked to the contrast-contrast phenomenon have been shown to operate *within* the primary visual cortex (V1) [9]. This suggests that both local, tuned suppression (such as mediated by somatostatin-containing inhibitory interneurons [10]), and long-range, V1-intrinsic inhibition (mediated by excitatory horizontal connections that target inhibitory interneurons) may be involved.

Reduced centre-surround interactions on perceived contrast have been replicated [11], [12] and have also been observed for the processing of motion [13], size [14], [15] and orientation [16]. The ubiquitous nature of gain control mechanisms in human visual processing means that it could provide a coherent framework for understanding the wide range of perceptual deficits observed in SZ [7].

In a similar vein, impaired *cognitive coordination* has also been put forward as a potential reason for deficits in the processing of visual context in SZ [17]. Cognitive coordination refers to those processes involved in modulating the salience of visual structure – e.g. through changes in the timing of neural signals – and it is manifest through phenomena labelled e.g. selective attention and, in particular, *grouping*. Grouping refers to the rules governing the perceptual association of simple local-features into more complex global-structures [18]. The balance of evidence suggests that patients with SZ have a deficit in visual grouping compared to unaffected controls (reviewed in [19]). In particular, patients with SZ have difficulty with tasks that require integration to reveal global spatial form [20], [21], [22] or global motion (for review see [23]) including biological motion [24]. This deficit can again lead to *superior* performance in SZ – for example, at ignoring the presence of irrelevant groupings when enumerating line segments [25] – ruling out a more generalised explanation.

**Figure 1:**
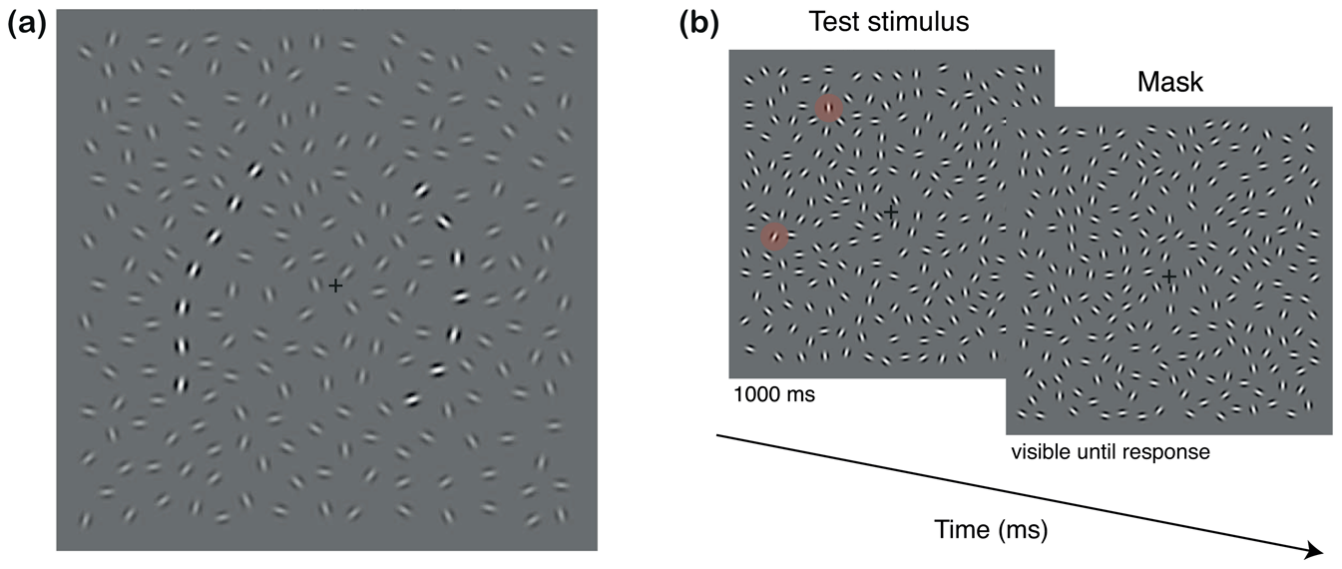
Stimuli and trial procedure from Experiment 1. (a) An example of the stimuli from the first experiment (with the contrast of distractors reduced for illustrative purposes). Observers had to identify which side of the image contained a structured contour. In this case the contour is surrounded by near-perpendicular elements, which generally enhance detectability. Note that the random path on the right was generated in essentially the same way as the structured contour – except that the orientation of path-elements was randomised prior to presentation. Because of this the orientation of distractors surrounding the random-path is comparable to the context of the structured contour in that elements are near-perpendicular to the contour-spine used to *generate* the random path. (b) A typical trial of Experiment 1: the test stimulus, which contained a structured contour either on the right or on the left (here the first and the last elements of the path are shaded in red to assist the reader in finding the contour) was immediately followed by a mask with randomly oriented elements. This display persisted until observers had made a response.

In this paper we focus on two tasks involving the perception of orientation. The first is c*ontour integration*: the linking of the oriented elements of a contour across space (for review see [26]). This is probed using a psychophysical paradigm where the observer must detect a contour composed of discrete oriented patches (Gabors), embedded in an array of randomly oriented distractor-elements [27] (see Figure 1a). This paradigm has been used to uncover the rules governing linkage; e.g. that it is tuned for the SF of elements [28], [29] and is much cruder in the peripheral visual field, apparently relying on the output of large spatial filters [30]. Furthermore, the immediate *context* that a contour arises in matters: observers have more difficulty finding contours embedded in distractors that are near-parallel than near-perpendicular to the local contour orientation [31], [32]. Contour integration paradigms have proven invaluable for probing the specific nature of the grouping deficit in SZ. Patients require closer spacing of elements to detect contours [14], [15], [22], [33] assessed using contour card system [34]. This deficit has been linked to a specific subtype of SZ characterised by thought disorder – as assessed using the *Positive and Negative Syndrome Scale* (PANSS) [35]. These deficits are particularly manifest for tasks where top-down cognitive control is required [33].

The collapse in our ability to see complex/curved contours in the periphery [30] relates to the second visual phenomenon we consider: *crowding*. Crowding refers to the disruptive effect of “clutter” (task-irrelevant flanking features) on our ability to recognise target-objects (for review see [36]). Crowding can affect our ability to determine the local orientation of features, with observers making reports that are consistent with the target-orientation having been averaged with the orientation of the flankers [37]. Crowding of orientation is more pronounced within contours [38] leading some to propose that crowding is contour grouping “gone awry” [38], [39], [40], [41]. Recently we have linked crowding to the effects of context on contour integration [32]. These results accord with the notion that spurious grouping of background-elements – with one another and with the contour-elements – is the primary limitation on contour grouping rather than the limits of a particular model *per se* [42]. In short the balance of evidence is that performance on contour integration tasks reflects an inter-play of limits set by visual integration (of contour-elements) and interactions of individual elements with their surrounding context. Such interactions can improve or interfere with contour localisation, with interference effects being in part attributable to crowding.

In light of these findings, the contour-grouping deficit in SZ has largely been attributed to differences in integration. In this paper we explore how abnormal processing of *visual context* may contribute to patients’ poor performance with tasks involving visual contour integration. We begin by assessing the ability of patients with SZ to localise contours in the presence of random variation in the local orientation of path-elements and how their performance is affected by the presence of contextual information that either helps or hinders performance in healthy controls [31], [32]. As well as replicating previous deficits in contour localisation we find that while perpendicular contexts facilitate localisation in both patients and controls, near-parallel contexts disrupt performance less in the clinical than in the unaffected group. These results are without doubt of great interest to the field of contour localisation; however, the most interesting findings of our study come from the second experiment, where we examined the effects of visual crowding on the processing of the individual contour-elements. Specifically, we explored the idea that the pattern of performance we observed in the first experiment could arise from differences in the way local elements of the stimuli are processed. Specifically we show that patients are poorer at reporting local orientation – of isolated Gabor elements – but show proportionally less crowding from flanking elements (i.e. they are less prone to interference from nearby distractors). Taken together these results indicate that differences in processing of surrounding context contribute significantly to the contour integration deficit in SZ. The influence of weaker contextual interactions could be direct – e.g. reduced ability to use context to localise contours – or indirect – e.g. leading to broader tuning for orientation in primary visual cortex, which in turn would reduce sensitivity to local orientation.

## Experiment 1: Contour localisation and sensitivity to context

Several studies have reported poor contour detection in patients with SZ [14], [15], [22], [33], [43], [44], [45], [46], [47], a deficit largely attributed to differences in integration. In the first experiment we tested the hypothesis that poor contour detection may be related to differences in the processing of context. To this end we measured the ability of observers with SZ to localise contours embedded in different contexts. Specifically, we assessed whether patients were affected by the presence of contextual information that either helped or hindered performance in healthy controls [31], [32].

### Methods and Materials

#### Ethics statement

To take part in this study all observers gave informed written consent in accordance with the Declaration of Helsinki. Ethics approval was granted by University College London’s local ethics committee.

#### Observers

Participants were 18 patients [12 males; mean age 39.2 years (σ = 8.0 years); mean IQ 104.3 (σ = 9.3) assessed with the *Revised National Adult Reading Test* (NART) [48]] diagnosed with schizophrenia (1 male and 2 females) or paranoid schizophrenia (15 patients). All were diagnosed independently of this study according to DSM-IV criteria and their clinical state was evaluated with the *Positive and Negative Syndrome Scale* (PANSS) [35]. Sixteen patients were treated with atypical antipsychotics, one with typical antipsychotics and one was unmedicated. The non-clinical control group comprised 12 male and 6 female participants recruited from university offices [mean age 40.7 years (σ = 9.4); mean IQ 109 (σ = 9.3)]. The two groups did not differ significantly for age (t_34_ = −0.51, p = 0.611) or for IQ (t_34_ = −1.51, p = 0.141).

#### Apparatus

Experiments were run on an Apple MacBook computer under the Matlab programming environment (MathWorks, Cambridge, MA) using software from the Psychophysics Toolbox [49]. Stimuli were presented on a LaCie Electron Blue 22″ CRT monitor and a 19″ Sony Trinitron Multiscan E400 monitor. Both monitors were calibrated with a Minolta photometer and linearized using custom-written software, giving a mean and maximum luminance of 50 and 100 cd/m^2^, respectively. In both cases the display resolution was 1024×768 pixels and the refresh rate was 75 Hz.

#### Stimuli

Test stimuli (Figure 1) consisted of contours composed of seven spatial-frequency band-pass Gabor micro-patterns (Gabors co-aligned with an underlying contour-spine), embedded in a field of distractor-Gabors [27]. The center-to-center separation of contour-elements was 56 arcmin and the whole stimulus subtended a 12.8×12.8 deg. square containing on average 220 elements (σ = 3.9 elements). All elements were in cosine phase, had a peak spatial frequency of 3.75 c/deg with an envelope σ of 5.7 arcmin, and were presented at 95% contrast.

Stimuli were generated as in Robol et al. [32]. In brief, we used standard contours (*snake*-contours) with a 15° path angle where the sign of the orientation difference between subsequent elements was randomised. As before, stimuli were manipulated so that contour-elements were clearly located in either the left or the right half of the image. This was achieved by forcing the middle contour-element to (a) pass through a region within ±0.53 deg. of the centre of a given image-half and (b) to have an orientation within ±45° of vertical. Further, no single contour-element could pass within 0.9 deg. of the edge of the image; nor could the contour cross itself. With these constraints, the average distance of the contours from fixation was ∼3.2 deg.

Stimuli were made by first inserting two contours – one in the left and one in the right half of the image – and then dropping distractor-elements on to the background. A minimum inter-element separation of 40 arcmin was maintained, thereby matching the mean-distance of any element – within contour or background – to its nearest neighbour. The orientation of distractor-elements was manipulated to obtain three surround conditions: *random*, *near-parallel* and *near-perpendicular* (Figure 1). We used the inverse of the Gaussian function (σ = 1.0 deg.) of the distance between distractors and contour-elements to set the orientation of distractor-elements – offset by 0° (near-parallel) or 90° (near-perpendicular). In the random condition (our baseline) the orientation of the distracting surrounding-elements was randomised.

At this stage of the stimulus generation procedure we have an image containing two contours, one on either side of fixation, for which the distractor-elements surrounding each have been subjected to the same contextual constraints (with respect to the contour on each side). We subsequently made our “random contour” by simply randomising the orientation of the elements within one of these contours. The observers’ task was then to report the side of the image containing the structured contour. Figure 1 shows an example (with the contrast of surrounds reduced for the purpose of illustration).

Prior to stimulus presentation we jittered the orientation of the elements within the structured contour. We did this by generating Gaussian random offsets with a standard deviation in the range 0–90° (note that this is the generating standard deviation – the true/wrapped standard deviation will be lower). A generating Gaussian standard deviation of 90° will produce a near-isotropic distribution of orientations. The level of orientation jitter was under control of an adaptive staircase procedure (QUEST [50]), as described in the *Procedure* section below. The orientation of distractor-elements was not modified further based on the new (noisy) contour orientation structure. Thus, in the near-parallel condition for example, the immediate surround was near-parallel to the contour-spine even if the orientation of each contour-element had been drastically altered.

Stimulus presentation was immediately followed by a mask composed of a field of randomly oriented elements (with on average the same number and separation of Gabors as the test stimulus). This display persisted until observers gave a response.

#### Design

The experiment had a within-subjects design. The independent variable was the orientation offset of the contour’s immediate context, defined as the mean orientation of the surrounding-elements relative to the contour-spine. We tested three levels of orientation offset: 0° (surrounding elements near-parallel to the contour-spine), 90° (surrounding elements near-perpendicular to the contour-spine), and random (surrounding elements randomly oriented). The dependent variable was the maximum orientation jitter along the contour-path supporting 75% correct contour localisation (*threshold orientation jitter*, see *Procedure*).

#### Procedure

Stimuli were viewed binocularly at a distance of 129 cm from the LaCie monitor and 116 cm from the Sony monitor. These distances were chosen to assure that, with both monitors, the whole stimulus subtended 12.8 deg. square. Observers fixated a centrally presented marker during presentation of test and mask stimuli. Observers were presented with a test stimulus (for a fixed exposure duration of 1000 ms) containing a structured and a random contour embedded within distractor-elements and located right and left of the fixation marker. This screen was immediately followed by a mask, which contained randomly oriented Gabors and remained on the screen until observers gave a verbal response to the question “Which side of the stimulus contained the contour?”. Observers were instructed to keep their eyes on the central cross, ignore the mask and indicate (verbally) for each test image if the contour was on the left or on the right. The experimenter recorded their response using the computer-keyboard. If observers had difficulties telling left from right we asked them to indicate the direction with their hand. Observers were asked to guess when not able to localise the contour. Visual feedback (the contrast-polarity of the fixation marker) indicated a correct or incorrect response.

We selected a relatively long fixed exposure duration of 1000 ms because pilot experiments revealed that the minimum exposure duration for experienced observers to perform contour localisation at 75% correct with high level of orientation jitter (∼15°) was around this value. The orientation variability along the contour-path was controlled by an adaptive staircase procedure (QUEST [50]) with correct and incorrect responses causing an increase and a decrease in orientation variability, respectively. The procedure converged on the orientation variability that led to 75% correct contour localisation. We refer to this measure as the *threshold orientation jitter*. Observers completed at least three runs of 135 trials each (45 trials per surround condition). In this way, for each observer we obtained the mean threshold orientation jitter in each surround condition over at least 135 trials. Each run comprised all three surround orientation conditions (random, near-parallel, near-perpendicular).

Before data collection every observer was provided with some static examples of the stimuli (some of which had the contour highlighted in BOLD in order to better visualise the type of contour they were asked to localise) and then completed a practice session with doubled exposure duration.

#### Statistical analysis

To test the effect of context on contour localisation and whether this was different in patients and healthy controls, we first carried out a repeated-measures analysis of variance on threshold-values (which are a measure of tolerance to orientation jitter), with *group* (patients, controls) as a between-subject factor and *condition* (random, near-parallel, near-perpendicular) as a within-subject factor. To examine whether patients with SZ showed less *inhibition* from the surround we then calculated log-ratios between thresholds with organized and random surrounds (i.e. log[near-parallel/random] and log[near-perpendicular/random]) and carried out a repeated-measures analysis of variance on these values, with factors *group* (patients, controls) and *condition* (near-parallel, near-perpendicular). P-values for all *post-hoc t*-tests have been corrected for multiple comparisons using the *Bonferroni* procedure and corrected p-values are reported. The alpha-value was set to 0.05 for all statistical tests.

### Results

**Figure 2:**
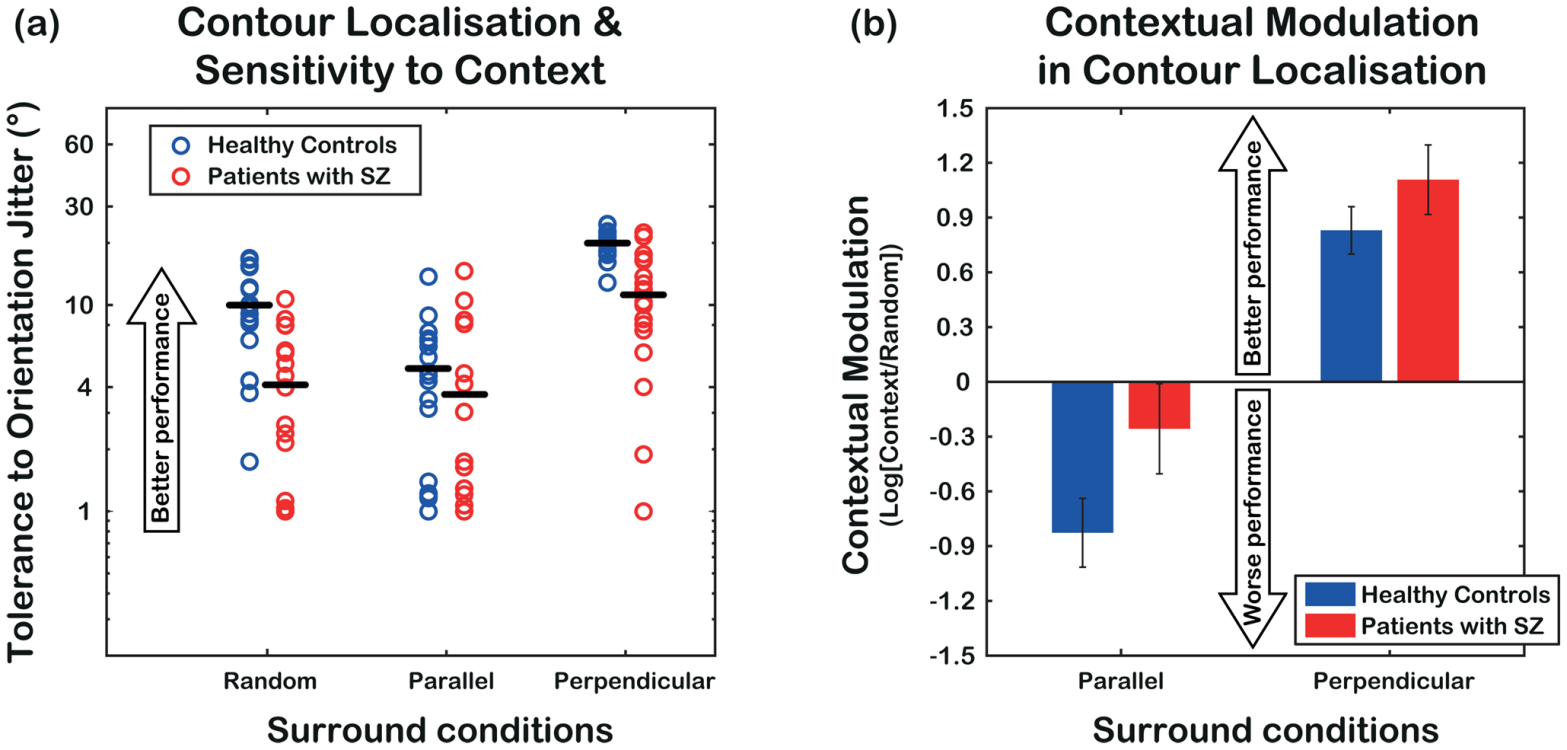
Results from Experiment 1. (a) Tolerance to orientation jitter for patients with SZ (red) and healthy controls (blue), measured with random, near-parallel and near-perpendicular surrounds. Black horizontal lines represent mean tolerance. Patients generally tolerate less orientation jitter than controls and are not affected by near-parallel contexts. (b) Log-ratios between tolerance with organized and random surrounds (i.e. log[near-parallel/random] and log[near-perpendicular/random]). Patients show less disruption from near-parallel surrounds compared to controls.

Figure 2a presents results from the first experiment for patients (red) and non-clinical controls (blue). Graphed data are *thresholds orientation jitter,* which were measured with random, near-parallel and near-perpendicular surrounds. Note that these thresholds represent a measure of tolerance to orientation jitter along the contour-path. This means that the higher the number the more orientation jitter observers tolerate and the better their performance. Analysis of variance on threshold-values shows a significant main effect of *group* (F_1,34_ = 22.73, p<0.001, two-tailed) and *condition* (F_2,68_ = 122.56, p<0.001, two-tailed) as well as a significant interaction (F_2,68_ = 12.73, p<0.001, two-tailed). Post-hoc comparisons show a significant difference between patients and controls only in the random (t_34_ = 4.34, p<0.001, two-tailed) and in the near-perpendicular surround conditions (t_34_ = 5.44, p<0.001, two-tailed). The finding that, in the baseline condition, (random surround) patients have lower thresholds than healthy controls (their tolerance is halved compared to controls: mean tolerance (± SE) is 4.11° ±0.74° vs. 9.99° ±1.14°) indicates generally poor contour localisation in patients. In addition, post-hoc comparisons indicate a different relative effect of near-parallel but not near-perpendicular surrounds in the two groups. Near-perpendicular surrounds increase tolerance (compared to the random surrounds) both in controls (19.97° ±0.69° vs. 9.99° ±1.14°, t_17_ = 10.03, p<0.001, two-tailed) and patients (11.23° ±1.45° vs. 4.11° ±0.74°, t_17_ = 6.10, p<0.001, two-tailed). On the contrary, near-parallel surrounds decrease tolerance in controls (4.91° ±0.78° vs. 9.99° ±1.14°, t_17_ = −5.03, p<0.001, two-tailed), but not in patients (3.69° ±0.96° vs. 4.11° ±0.74°, t_17_ = −0.453, p>0.05, two-tailed).

These data may suggest the presence of less *inhibition* from the surround (which may also be consistent with increased *facilitation*) in patients with SZ compared to healthy controls. To examine this prediction we performed a repeated-measures analysis of variance on log-ratios between thresholds with organized and random surrounds (as described in the *Methods* section). Note that a log-ratio of 0 indicates no effect of organized surround, whereas a log-ratio <0 indicates worse performance with organized than random surrounds (consistent with increased *inhibition* from the surround), and a log-ratio >0 reflects better performance in the presence of organized than random surrounds (consistent with increased *facilitation*).

As shown in Figure 2b and confirmed by the ANOVA results, in both groups log-ratios with perpendicular surrounds are positive and higher than with parallel surrounds (significant main effect of the factor *condition*, F_1,34_ = 114.94, p<0.001, one-tailed), consistent with facilitation from perpendicular surrounds. Additionally, log-ratios are generally higher in patients than controls (significant main effect of the factor *group*: F_1,34_ = 3.26, p = 0.040, one-tailed). No significant interaction was observed (F_1,34_ = 1.08, p = 0.153, one-tailed), consistent with performance in the two surround conditions being affected in the same way by the factor *group*.

Note that the pattern of contextual modulation in our patient group is not correlated to disorganization symptoms. Indeed, we found no correlation between disorganization (expressed as *cognitive factor*, see [20], [21], [22]) and contextual modulation indices (log-ratios), neither for the parallel surround (r_16_ = −0.389, p = 0.444 corrected for multiple comparisons, two-tailed) nor for the perpendicular condition (r_16_ = −0.311, p = 0.840 corrected for multiple comparisons, two-tailed).

Taken together, results for the control group confirm our previous findings [31], [32], showing facilitation (higher tolerance) from near-perpendicular surrounds and suppression (lower tolerance) from near-parallel surrounds. The pattern of results for patients indicates poor contour localisation and an *abnormal* processing of context. Indeed, although patients are poor at localising the contour embedded in random surrounds, they are also proportionally less disrupted by the presence of near-parallel surrounds than healthy controls. It is not the case that observers with SZ are generally less influenced by any contextual information since they exhibit as much facilitation by near-perpendicular surrounds as healthy controls (log[near-perpendicular/random] ratios (± SE) are 1.11±0.19 and 0.83±0.13, respectively, t_34_ = 1.20, p = 0.118 *Bonferroni*-corrected, one-tailed). Consistent with earlier findings [25] we find that differences in context-processing in SZ can impact on form detection in a positive way: in parallel surround conditions we see less disruption in the observers with SZ. A reduced influence of parallel surrounds is generally consistent with earlier reports of reduced surround suppression [7], [11], [12], [13], [14], [15] although earlier results focused on the processing of contrast.

Previously Robol et al. [32] have shown that the disruptive effect of near-parallel surrounds may in part be attributed to contours (frequently) falling in the peripheral field where recognition is prone to *visual crowding* (the disruptive effect of clutter on object recognition). Because crowding is widely believed to involve integration processes “gone awry” [51], if patients’ poor contour detection in Experiment 1 did arise from poor integration we might expect that they should be less affected (*crowded*) when the distractors formed contours with the target (since less integration should benefit observers under this condition). Under this view we should observe normal levels of crowding when distractors do not form contours with the target. Experiment 2 directly examines this prediction, by measuring orientation discrimination in isolated and crowded stimuli. Note that by making measurements of patients’ ability to discriminate the orientation of individual Gabors – presented under similar viewing conditions to the Gabor elements comprising the stimuli from Experiment 1– we are also able to ascertain if local orientation processing, and contextual influences, are affected in SZ.

## Experiment 2: Local processing of orientation in isolated and crowded stimuli

In Experiment 1 we showed poorer contour detection performance and less susceptibility to the influence of surrounding context in patients with SZ. In Experiment 2 we investigated local processing (of individual oriented element) in order to determine:

- If the reduced contextual processing found in Experiment 1 extends to local orientation processing.
- If generally poor contour detection in SZ arises not from a failure of grouping (i.e. *global* processing) but from poorer *local* encoding of the elements that make up these stimuli.
- If notionally poorer contour-grouping by patients with SZ leads to a selective reduction in crowding within contours.

To this end, we measured observers’ ability to discriminate if the orientation of a single Gabor element was presented clockwise or anticlockwise of vertical. Gabors were presented either in isolation (to give us baseline performance), or under crowded conditions comparable to the way the Gabor appeared in the contour experiments. We ran two crowded conditions with the target Gabor either (a) flanked on either side by a randomly oriented element or (b) flanked above and below by Gabors whose orientations formed a contour with the target Gabor. These two conditions sought to quantify the likely role in crowding played by the randomly oriented surround (the context) and by the local contour structure. Stimuli were of a similar size, eccentricity and (where applicable) spacing, to the contour-elements in Experiment 1.

### Methods and Materials

#### Observers

Thirteen of the observers with SZ (and their matched healthy controls) of Experiment 1 also participated in Experiment 2.

#### Apparatus

We used the same apparatus and display parameters as in Experiment 1.

#### Stimuli

**Figure 3:**
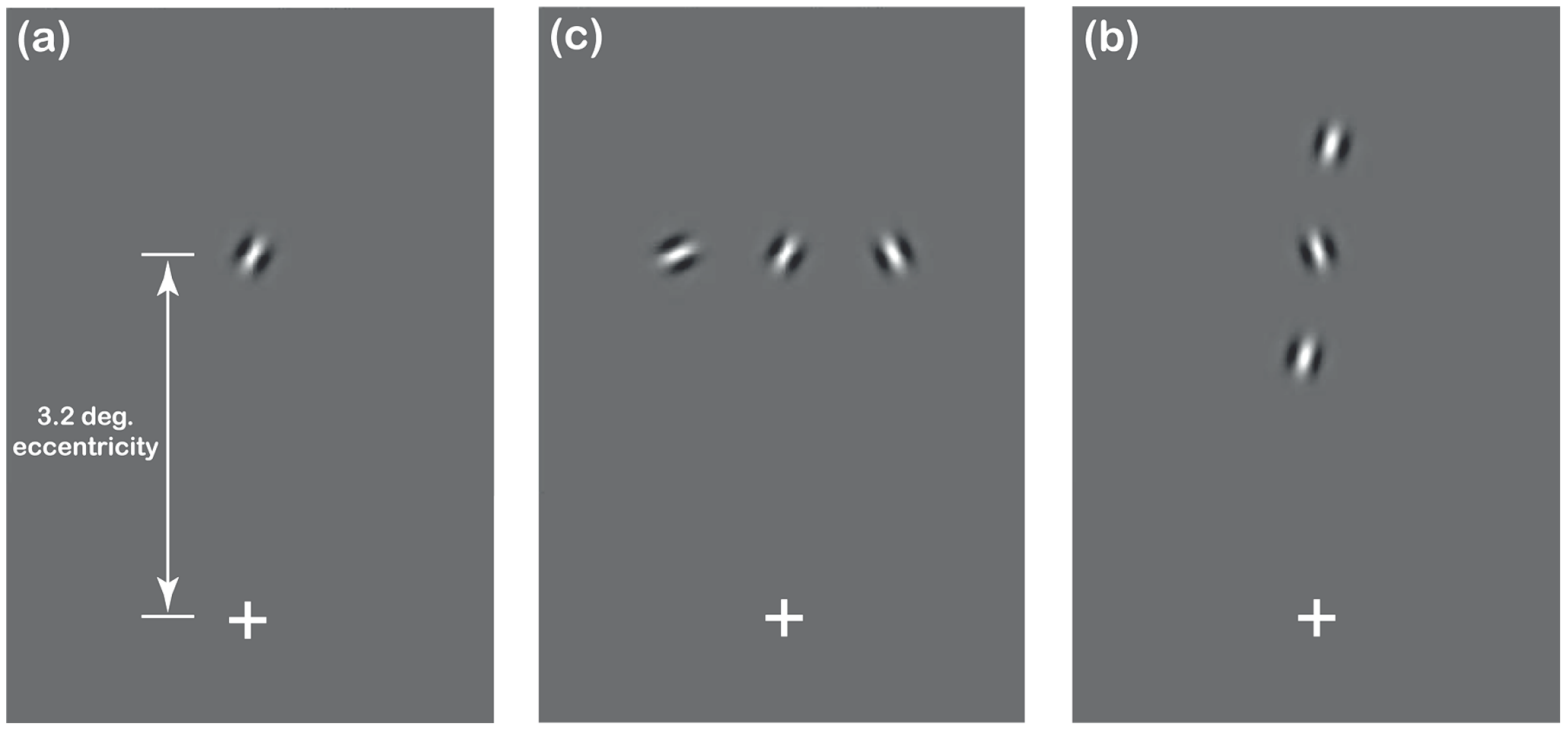
Examples of the stimuli from the second experiment, where observes had to judge the orientation of the central Gabor (clockwise or anticlockwise of vertical). (a) Isolated target condition. (b) Random flankers condition. (c) Contour-fragment condition.

In Experiment 2 we used Gabors with the same parameters as those in Experiment 1 (cosine phase, peak spatial frequency = 3.75 c/deg, envelope σ = 5.7 arcmin, 95% contrast). The target for the orientation judgement (clockwise or anticlockwise of vertical) was a Gabor presented in the parafovea (either upper or lower side of the screen, 3.2 deg. eccentricity), with or without similar flankers (Figure 3). Note that the average distance of the target from fixation was matched in the two experiments (3.2 deg. eccentricity).

We tested three conditions: *isolated Gabor*, *random flankers*, *contour-fragment*. In the *isolated Gabor* condition, the target element was presented at an eccentricity of 3.2 deg. either above or below the fixation cross. When there were flankers (*random flankers* and *contour-fragment* conditions), separation of the Gabor elements was 56 arcmin (so matching the contour-elements’ separation used in Experiment 1). In the random flankers condition two randomly oriented elements (with similar spatial frequency/envelopes) flanked the isolated Gabor. Flankers were on the same horizontal axis as the target Gabor. In the contour-fragment condition we added two Gabors laying on a contour-spine defined using a vertical target orientation, thus forming a contour-fragment (vertically oriented). In this way flanker-orientation and position were not informative of the target orientation. Path angle was 15°, with the sign of the orientation difference between subsequent elements randomised.

We manipulated the target tilt (clockwise or anticlockwise of vertical), pre-selecting, for each flanker-condition, seven appropriate tilt values to fit psychometric functions (see below). For each condition, these values were selected based on pilot data that indicated they bracketed the whole psychometric function (and not just a part of it) for observers tested under that specific condition of crowding. Note that this inevitably resulted in different tilt-values in the three flanker-conditions (indeed the strength of crowding is not the same in the three conditions tested).

#### Design

We used a within-subjects design and tested three conditions: (i) isolated target, (ii) target plus 2 randomly oriented flankers, (iii) contour-fragment. In each condition the independent variable was the degree of tilt of the target set according to a method of constant stimuli (MOCS) with seven levels: −6°, −4°, −2°, 0°, +2°, +4°, +6° (in the isolated Gabor condition), −9°, −6°, −3°, 0°, +3°, +6°, +9° (in the random flankers condition) and −45°, −30°, −15°, 0°, +15°, +30°, +45° (in the contour-fragment condition). The dependent variable was the probability to report that the target was tilted clockwise of vertical.

#### Procedure

As in Robol et al. [32], stimuli were viewed monocularly (with observers’ dominant/sighting eye). This increased the difficulty of the task, thus reducing the probability of ceiling performance. Viewing distance was as in Experiment 1. Observers fixated a centrally presented marker (a white cross) during presentation of the test stimulus, which appeared peripherally either in the upper or in the lower half of the screen (3.2 deg. eccentricity). Stimuli were presented for 125 ms. Observers were required to fixate a central white cross throughout the whole experiment. Observers indicated (verbally) whether they thought the central striped “blob” (the target) was tilted clockwise or anticlockwise of vertical, and the experimenter recorded their response using the computer keyboard. Visual feedback (the contrast-polarity of the fixation marker) indicated a correct or incorrect response. Observers were provided with some static examples of the stimuli in the information sheet. To verify that the task was correctly understood, we first allowed observers to look directly at the stimuli (instead of at the fixation cross). Then we ran a practice session where all target tilts were doubled (so that in most cases it was obvious whether the central Gabor was tilted to the right or to the left). In the practice session we checked the observers’ response trial-by-trial, gave verbal feedback and, when necessary, re-presented the stimulus and explained why the response was not correct. If observers had difficulty telling left from right we asked them to indicate the direction with their hand.

Three conditions (each comprising seven target tilt levels) were interleaved in a single run. Observers completed at least one run of 336 trials each (3 conditions × 7 levels per condition × 16 trials per tilt level). Raw data were fit with cumulative Gaussian functions (assuming a fixed lapse rate of 5% for both patients and matched controls in all flanker-conditions, but see the *Appendix S1* for a control of the lapses of attention), to give an estimate of response-variance (threshold) and bias (point of subjective equality; PSE). Since there was no difference in correct responses for the upper and lower sides of the screen – for both patients (t_14_ = −0.02, p = 0.981, two-tailed) and healthy controls (t_14_ = −0.63, p = 0.540, two-tailed) – raw data were fit independently of stimulus position. There were no systematic trends in PSE data – both clinical and non-clinical groups were uniformly unbiased – and we do not consider these data further.

#### Statistical analysis

To compare the effect of flankers in patients and controls we first carried out a repeated-measures analysis of variance on threshold-values, with *group* (patients, controls) as a between-subjects factor and *condition* (isolated target, random flankers, contour-fragment) as a within-subjects factor. We then estimated the amount of crowding from random flankers and within contours in each group by calculating log-ratios between thresholds in the crowded and isolated stimuli (i.e. log[random/isolated] and log[contour/isolated]). To compare the amount of crowding in patients and controls and test the prediction that patients should show less crowding we performed a repeated-measures analysis of variance on log-ratios, with *group* (patients, controls) as a between-subject factor and *condition* (random flankers, contour-fragment) as a within-subject factor. The *Bonferroni* procedure has been used to correct p-values for multiple comparisons. Alpha-value was set to 0.05 for all statistical tests.

### Results

**Figure 4:**
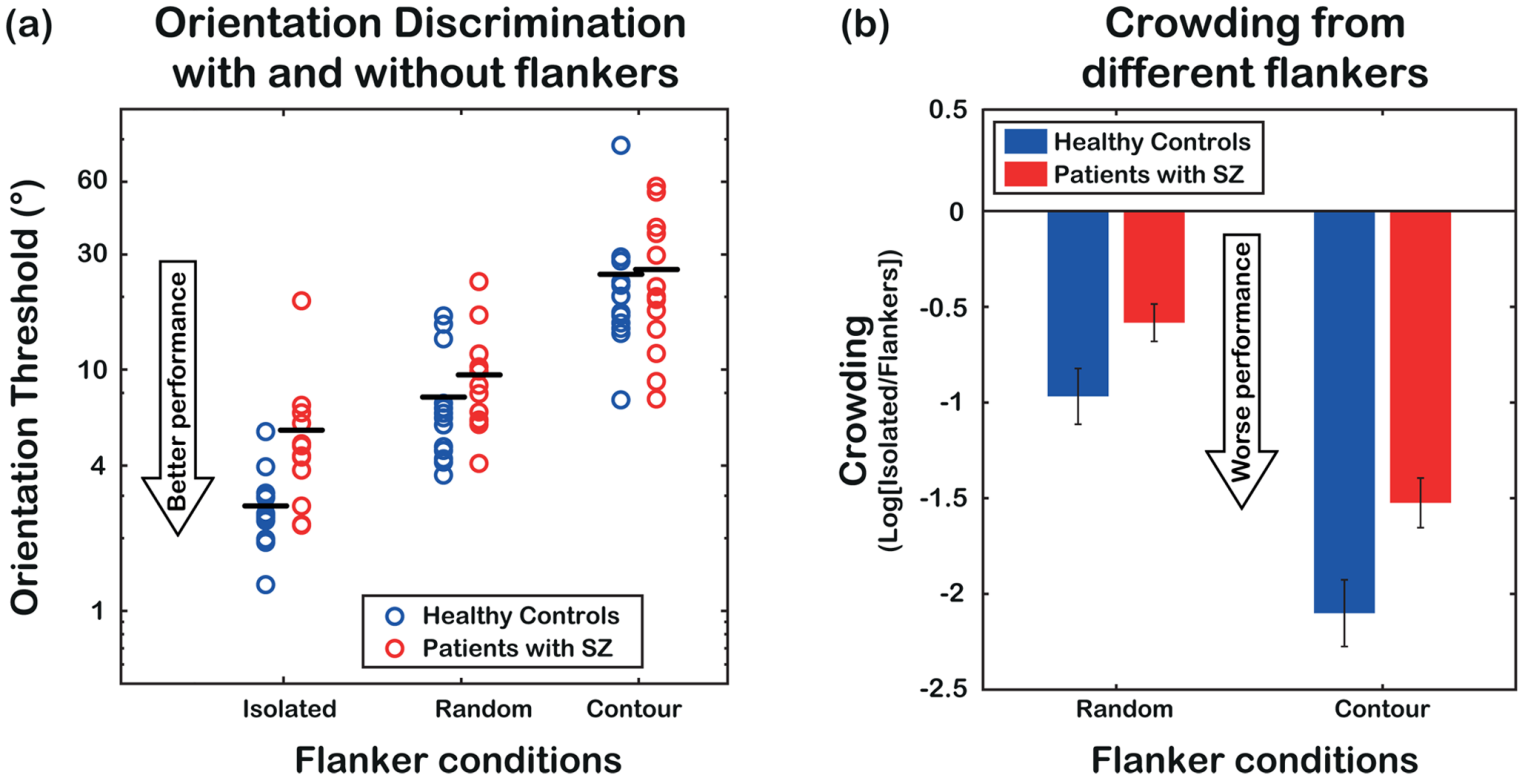
Results from Experiment 2. (a) Mean orientation discrimination thresholds for patients with SZ (red) and non-clinical controls (blue) in the three conditions tested in Experiment 2 (isolated Gabor, random flankers, contour-fragment). Black horizontal lines represent mean orientation thresholds. Note that in this graph higher y-values indicate *poorer* performance, whereas in Figure 2a higher y-values indicate *better* performance. Patients’ thresholds in the isolated-Gabor condition are doubled compared to controls’, indicating reduced sensitivity to local orientation. (b) Log-ratios between thresholds in the isolated and crowded stimuli (i.e. log[isolated/random] and log[isolated/contour]). Both in the random flankers condition and in the contour-fragment condition patients show less crowding compared to controls.

Figure 4a presents mean orientation discrimination thresholds for patients (red) and non-clinical controls (blue) in the three conditions tested in Experiment 2 (isolated Gabor, random flankers, contour-fragment). We note that patients are poor at discriminating the orientation of an isolated element: thresholds are indeed doubled compared to controls (mean thresholds (± SE) are 5.61° ±1.21° vs. 2.73° ±0.30°, t_24_ = −2.31, p = 0.03). This indicates poor processing of local structure (the constituents of contours). Analysis of variance on threshold-values, with *group* (patients, controls) as a between-subject factor and *condition* (isolated target, random flankers, contour-fragment) as a within-subject factor, indicates a significant effect only for the main factor *condition* (F_2,48_ = 36.20, p<0.001, two-tailed). That the *group x condition* interaction is not significant suggests that adding flankers increases thresholds both in healthy controls and in patients.

To quantify the amount of crowding from randomly oriented flankers and contour-consistent flankers, we calculated log-ratios between orientation thresholds in the crowded and isolated stimuli (i.e. log[random/isolated] and log[contour/isolated]). The mean log-ratios for patients and controls are presented in Figure 4b (note that in this figure we present log[Isolated/Flankers] in order to better compare graphically these results to those of Figure 2b). Analysis of variance on log-ratios, with *group* (patients, controls) as a between-subjects factor and *condition* (random flankers, contour-fragment) as a within-subjects factor, shows a significant main effect of group (F_1,24_ = 9.16, p = 0.003, one-tailed) and condition (F_1,24_ = 78.11, p<0.001, one-tailed) with no significant interaction (F_1,24_ = 0.67, p = 0.211, one-tailed). This indicates that patients show lower log-ratios compared to healthy controls both in the condition where the target is flanked by two randomly oriented elements and within contours. Note that this pattern of results does not reflect a ceiling effect. Indeed, in a control experiment on one healthy observer we measured a threshold in the contour-fragment condition that was 6 times higher than in the isolated Gabor condition (16.64° vs. 2.65°). With noise superimposed on the stimuli – to elevate the baseline threshold and thus mimic patients’ performance in the isolated Gabor condition – we could still observe a 6X threshold-increase (37.09° vs. 5.89°). This shows that our paradigm was not limited in its ability to estimate the effect of crowding because of some elevation in baseline performance in patients.

This pattern of results is not the consequence of patients having generally performed poorer e.g. as a result of inattention. If it were, we would expect patients to exhibit systematically higher lapse rates (i.e. stimulus-unrelated errors made with “easy” stimuli within the tails of the psychometric function). In the *Appendix S1*, we show that the overall mean lapse rate of patients and controls do not differ systematically (Figure S1) and, importantly, we get the same pattern of effects as reported here (i.e. less crowding in patients compared to controls) when fitting using the (group) mean lapse rate for each condition.

Taken together, the results of Experiment 2 indicate both reduced sensitivity to local orientation and relatively weaker crowding in observers with SZ, confirming our prediction that they should be less affected by the disruptive influence of distractor-elements on object recognition in the periphery. Additionally, these results suggest a role of poor local processing in the contour localisation deficit shown by patients with SZ.

Note that, at least for the contour-fragment condition, we cannot rule out a role of the clinical state in the reduced crowding shown by patients. Indeed, we found a significant negative correlation between the total score of the PANSS Negative Scale and the effect of flankers (log-ratios) in the contour-fragment condition (r_11_ = −0.684, p = 0.040 corrected for multiple comparisons, two-tailed). In other words, patients who scored more highly on the PANSS Negative Scale experienced less crowding from flankers within contour. Note that no other PANSS scores correlated significantly with performance on tasks in this experiment or in Experiment 1.

## Discussion

In Experiment 1 we measured the ability of observers with SZ to localise a contour embedded in different surrounds (random, near-parallel and near-perpendicular). We reported that, although patients were poorer at detecting contours embedded in random noise, they were proportionally less disrupted by the presence of near-parallel surrounds than healthy controls. Conceptually, these results are consistent with earlier reports of reduced surround suppression in SZ [7], . We then measured the ability of observers with SZ to discriminate the orientation of the local components of our contours (Experiment 2) and showed that although patients performed worse at this task (i.e. orientation thresholds were higher), they were less affected by the disruptive influence of distractor-elements (i.e. they were less prone to visual crowding).

### Orientation Discrimination, Gain Control, GABA and NMDA-dysregulation

*Gain control* refers to the inhibitory cortical processes that allow neurons to optimise their limited operating range (for a recent review see [52]). We can distinguish between at least four gain control mechanisms: (i) local untuned suppression (such as mediated by parvalbumin-containing interneurons [10]); (ii) local tuned suppression (such as mediated by somatostatin-containing interneurons [10]); (iii) long-range, V1-intrinsic inhibition (mediated by excitatory horizontal connections that target inhibitory interneurons) and (iv) long-range, feedback inhibition (mediated by excitatory projections targeting local inhibitory neurons).

It has been proposed that gain control within V1 plays a substantial role in the *contrast-contrast* illusion. Dakin et al. [7] have proposed that their finding that patients with SZ are less prone to this illusion could be a consequence of reduced gain control. A reduction in these centre-surround interactions in SZ has been widely reported for motion processing [13], for the processing of size [14], [15] and recently also for orientation [16]. Cortical levels of γ-aminobutyric acid (GABA) – the chief inhibitory neurotransmitter in humans – is thought to play a crucial role in these centre-surround interactions, not only through local suppression (both tuned and untuned [10]), but also through inhibitory long-range intra-cortical, horizontal connections as well as long-range feedback projections [53], [54], [55].

GABA cortical levels are lower in SZ [56], [57] and also correlate with the amount of visual surround suppression measured psychophysically [56]. Data from studies of humans and non-human primates suggest a role of GABA-mediated inhibition also in orientation discrimination (although see [58], [59], which show that cortical inhibition may not be necessary for orientation tuning). Physiological reports show that GABA-mediated inhibition modulates neuronal selectivity in the visual system [60] and specifically the selectivity of visual cortical neurons to stimulus orientation [61], [62], [63], [64], [65], [66]. The specific action of GABA blockage (e.g. via administration of Gabazine) seems to be to elevate overall levels of activation of neurons [65]; some authors have interpreted this as a broadening of tuning while others have proposed that tuning is essentially unchanged but that response now sits on top of a pedestal of higher underlying spontaneous activity. In terms of human data, the importance of GABA-levels for orientation discrimination has recently been confirmed using magnetic resonance spectroscopy [67] and preliminary evidence [68] indicates a negative correlation between human visual cortical levels of GABA and human orientation discrimination performance.

A reduction in orientation-selectivity of individual neurons due to decreased GABA-mediated inhibition (specifically, via local tuned connections, long-range, V1-intrinsic interactions and feedback processes) could account for the poorer local orientation discrimination performance (relative to controls) we reported in Experiment 2.

However, benzodiazepines, which directly enhance the effects of GABA at the receptor level, do not ameliorate psychotic symptoms [69]. A putative *indirect* route for GABAs action in SZ is through its facilitatory effects on dopamine (DA) pathways. GABA interneurons are known to modulate the mesolimbic DAergic system, which is directly implicated in the positive symptomology of SZ [70]. Further, drugs that enhance the effects of DA have been shown to mimic many of the symptoms of psychosis [71], [72], and the success of antipsychotic drugs has been directly linked to their affinity for DA receptors [73], [74]. In addition, GABA may either have an inhibitory or facilitatory effect on DA, depending on local concentrations and pre-existing levels of activity [75], [76], [77] (see [78] for a discussion also). One could hypothesise that such a contingent effect could underlie the differences in performance on tasks that have been linked to cortical and pre-cortical structures [16]. In support of this possibility, there have been claims of co-existing sub-cortical *hyper-*dopaminergia (an excess of DA) and cortical *hypo-*dopaminergia (a deficit of DA) in SZ [79], [80] (see [81] for a related model of SZ).

Poor orientation discrimination could potentially be related also to the extensively reported N-methyl-D-aspartate (NMDA) receptor dysregulation in SZ (for a review see [82]). NMDA receptors, indeed, seem to play a critical role in gain control mechanisms. Several neurophysiological studies and animal models have shown that NMDA-receptors amplify the responses to isolated stimuli and increase the effects of lateral inhibition (for a review see [83]). In the light of these results, NMDA-receptor dysregulation likely results in less amplification and decreased lateral inhibition. An indication of decreased signal amplification in patients with SZ comes from the study by Butler et al. [5], who reported that patients’ visual evoked potential contrast response curves show decreased gain at low contrast as well as a lower plateau. Interestingly, studies on NMDA-receptor activity in cat visual cortex and lateral geniculate nucleus [84], [85] have reported similar effects (i.e. decreased gain at low contrast and lower plateau), suggesting a substantial role of NMDA-receptor in gain control.

### The Role of Inhibition in Contour Integration

A deficit in a circuitry that, from a computational point of view, is *inhibitory* is suggested not only by the reduced local orientation discrimination in patients (Experiment 2), but also by the reduced disruptive effect of near-parallel surrounds on their ability to localise contours (Experiment 1). This finding is indeed consistent with less suppression from iso-oriented distractor-elements in the background. If, as suggested by Chapman and Chapman [86], patients have problems in ignoring irrelevant stimuli, they should have particular problems with near-parallel surrounds (that could be characterised as presenting more plausible alternatives to the contour). We report the opposite: patients are better at ignoring such disruptive surrounds. Thus, the result that patients are relatively good in this condition cannot be accounted for by a general inability to ignore irrelevant stimuli but must be attributable to a more specific deficit that we propose is related to dysfunctional cortical inhibition.

The crucial role of inhibition in contour integration has been emphasized in Yen and Finkel’s [87] model. In this cortical-based model, contour integration reflects the level of synchronization of activity of units responding to inter-related contour-segments, which strongly depends on the balance of facilitatory and inhibitory inputs from contour- versus background-elements. In a first stage two sets of *facilitatory* connections operate, the *co-axial* and the *trans-axial* connections, which run parallel and orthogonal to the local contour direction, respectively. After co-axial and trans-axial patterns of activity around a given point in space have been compared, *inhibitory* connections switch off the responses of all those units whose facilitation from other active cells falls below a given threshold. Finally, strongly facilitated units undergo temporal synchronization, with the sum of the activity of all synchronized units determining the perceptual salience of the contour.

In this framework the disruptive effect of near-parallel surrounds in healthy controls (Experiment 1) likely reflects inhibitory inputs from iso-oriented surrounding elements (consistent with surround suppression from iso-oriented distractors). Decreased inhibitory inputs from iso-oriented elements in the immediate surround would account for the reduced influence of near-parallel surrounds in SZ. Note that reduced inhibition can also account for patients’ poorer localisation of contours in random surrounds. Two aspects of the Yen and Finkel’s [87] model are relevant in this regard: (i) the importance of the balance between facilitation and inhibition for contour integration and perceived contour salience and (ii) the fact that facilitation and inhibition operate in parallel over the scene and extract not only the target-contour, but also, other less salient contours. A reduced inhibition in SZ would lead patients to perceive more spurious contour-fragments arising in the background by chance. An inability to ignore these irrelevant contour-structures in the random-noise [86] would make them vulnerable to lots of “false alarms” in the background. This could also potentially predict increased susceptibility to hallucinatory experiences in noise – abnormal sensory experiences related to the loss of distinction between relevant and irrelevant stimuli [88], [89], [90].

### The Role of Inhibition in the Reduced Crowding in SZ

Recent findings suggest that the attributes (e.g. orientation or position) of local stimuli in crowded displays are *averaged* or *pooled* [37], [39], [91]. For example, observers generally make reports that are consistent with the target-orientation having been averaged with the orientation of the flankers [37]. Crowding is stronger (i.e. pooling is more pronounced) within contours than within other arrangements [38], which led to the proposal of a close link between crowding and contour grouping [38], [39], [40], [41]. Given this and the known visual grouping deficit in SZ (reviewed in [19]), then if crowding *only* involved pooling patients should have shown more release from crowding (compared to controls) in the contour-fragment condition compared to the random-flanker condition (which they did not). An alternative interpretation consistent with our results is that crowding also relies on inhibitory local interactions between spatially adjacent mechanisms selective to similar visual features [92], [93], [94], [95]. Our results suggest that this local, tuned suppression (such as mediated by somatostatin-containing inhibitory neurons [10]), but also more spatially extended V1-intrinsic inhibitory connections may be affected in SZ.

### Conclusion

In conclusion, our data of Experiment 1 are consistent with reduced suppression rather than a general decrease in all contextual effects on contour localisation in SZ. The poor local orientation discrimination and the reduced crowding in SZ (Experiment 2) also are consistent with a reduction in inhibitory V1-intrinsic interactions. We suggest that this pattern could result from abnormal gain control, which is crucial both in orientation-selectivity and in surround suppression. The association of reduced crowding with greater levels of negative symptoms in patients with schizophrenia suggests that pharmacological compounds able to specifically modulate gain control may be potential biomarkers of novel treatments for negative symptoms, which remain largely untreated with current antipsychotic medication.

## Supporting Information

Figure S1 **Best fitting lapse rates for patients (red circles) and matched controls (blue circles) in the three conditions tested in Experiment 2 (i.e. isolated target, random flankers, contour-fragment).** The overall mean lapse rate of patients and controls do not differ systematically.
(TIF)

Appendix S1 **Control of the lapses of attention in Experiment 2: method and results.**
(DOC)
