# Correction: Reduced Crowding and Poor Contour Detection in Schizophrenia Are Consistent with Weak Surround Inhibition

**DOI:** 10.1371/journal.pone.0218483

**Published:** 2019-06-13

**Authors:** Valentina Robol, Marc S. Tibber, Elaine J. Anderson, Tracy Bobin, Patricia Carlin, Sukhwinder S. Shergill, Steven C. Dakin

The following information is missing from the Funding statement: This study was supported by the European Research Council Executive Agency (ERC).
