# Supplementary figures and images for: Reduced Crowding and Poor Contour Detection in Schizophrenia Are Consistent with Weak Surround Inhibition
Source: PLoS One. 2013 Apr 9;8(4):e60951. doi: 10.1371/journal.pone.0060951 (PMC3621669; doi:10.1371/journal.pone.0060951)

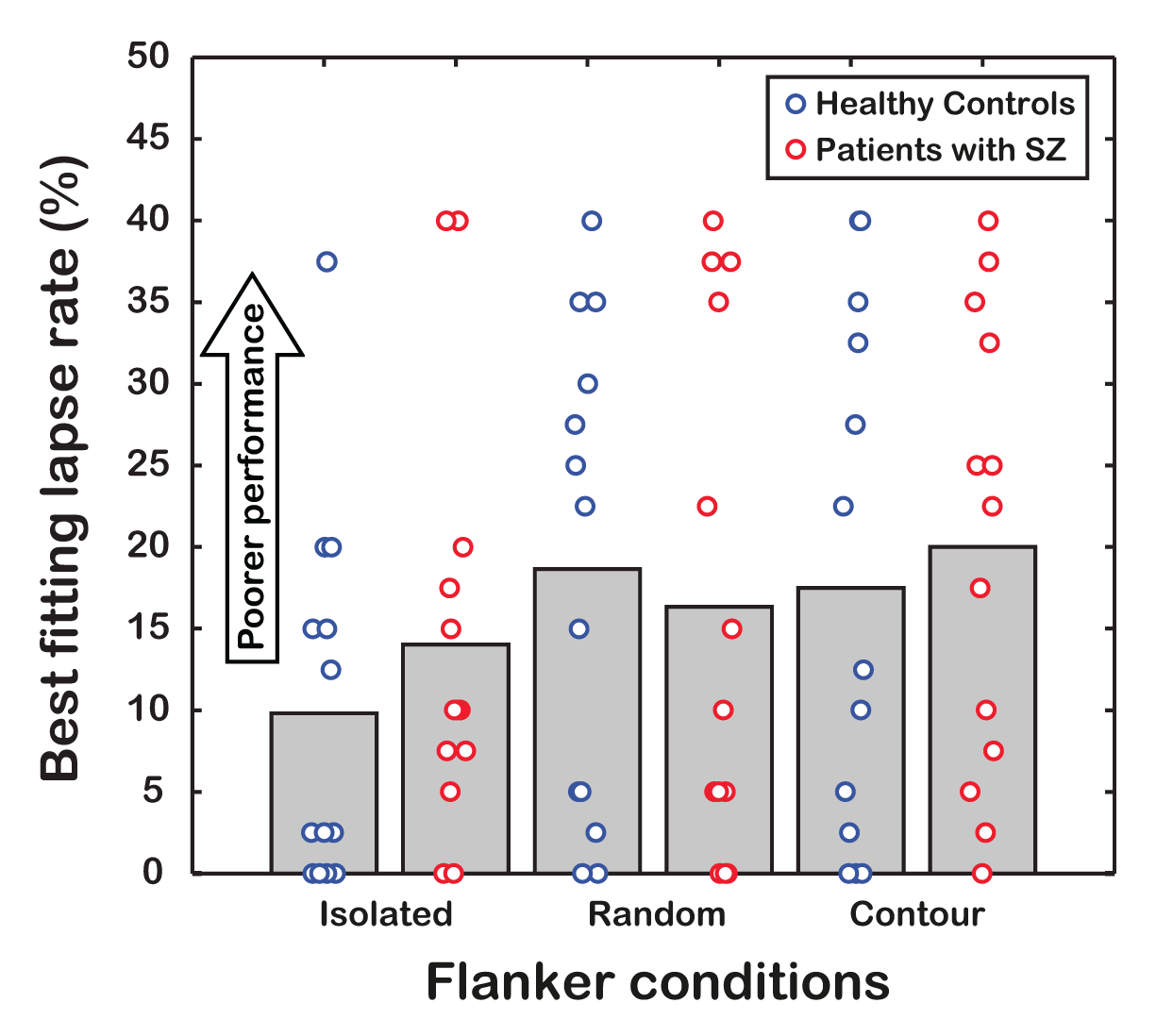

Supplement: Figure S1 — Best fitting lapse rates for patients (red circles) and matched controls (blue circles) in the three conditions tested in Experiment 2 (i.e. isolated target, random flankers, contour-fragment). The overall mean lapse rate of patients and controls do not differ systematically. (TIF) [file pone.0060951.s001.tif]
