# Supplementary material for: Reduced Crowding and Poor Contour Detection in Schizophrenia Are Consistent with Weak Surround Inhibition
Source: PLoS One. 2013 Apr 9;8(4):e60951. doi: 10.1371/journal.pone.0060951 (PMC3621669; doi:10.1371/journal.pone.0060951)
Supplement: Appendix S1 — Control of the lapses of attention in Experiment 2: method and results. (DOC) [file pone.0060951.s002.doc]

**Appendix S1**

Figure S1 presents the mean best fitting lapse rates for patients (red circles) and matched controls (blue circles) in the three conditions tested in Experiment 2 (i.e. isolated target, random flankers, contour-fragment). Best fitting lapse rates were derived by performing a series of two-parameter (i.e. bias and standard deviation) fits each with a different (fixed) lapse rate and then picking the lapse rate that minimised the least-squares difference between predicted psychometric function and the data, across all fits. Using the data so derived we then carried out a repeated-measures ANOVA, treating each pair of (matched) control and patient group data points as if they were two values recorded from the same observer (i.e. same idea as a paired sample t-test). In this way, we had two factors, both *within*: *group* (patients vs. controls) and *condition* (isolated target, random flankers, contour-fragment). Results show no main effect of the factor *group* (F_1,12_= 0.15, p = 0.704), nor of the factor *condition* (F_2,24_ = 2.87, p = 0.076), together with a non-significant *group x condition* interaction (F_2,24_ = 0.40, p = 0.675].

Most interestingly, our assumption about the lapse rate (i.e. 5% for both patients and matched controls in all flanker-conditions) did not determine the pattern of results we reported in Experiment 2. Indeed, for each observer in each condition we reran the fittings using the mean best fitting lapse rates across observers (within group, i.e. ~14%, ~16% and ~20% for patients and ~10%, ~19% and ~18% for controls in the isolated target, random flankers and contour-fragment conditions, respectively – see Figure S1). Although thresholds were now generally lower than before (for both patients and controls), the ANOVAs on raw thresholds and on log-ratios (as done before, see par. *Statistical analysis* of Experiment 2) confirm the pattern of results we reported in Experiment 2. Specifically, for raw thresholds again we report a significant effect only for the main factor *condition* (F_2,48_ = 22.71, p < 0.001, two-tailed). For log-ratios again we report a significant effect of the factors *group* (F_1,24_ = 4.77, p = 0.019, one-tailed) and *condition* (F_1,24_ = 40.41, p < 0.001, one-tailed) together with a non-significant interaction (F_1,24_ = 1.80, p = 0.096, one-tailed). This pattern of results confirms the presence of relatively weaker crowding in patients with SZ compared to matched controls.
